# Sessile serrated adenomas with dysplasia: morphological patterns and correlations with MLH1 immunohistochemistry

**DOI:** 10.1038/modpathol.2017.92

**Published:** 2017-07-28

**Authors:** Cheng Liu, Neal I Walker, Barbara A Leggett, Vicki LJ Whitehall, Mark L Bettington, Christophe Rosty

**Affiliations:** 1 The Conjoint Gastroenterology Laboratory, QIMR Berghofer Medical Research Institute, Brisbane, QLD, Australia; 2 Faculty of Medicine, University of Queensland, Brisbane, QLD, Australia; 3 Envoi Specialist Pathologists, Brisbane, QLD, Australia; 4 The Royal Brisbane and Women’s Hospital, Brisbane, QLD, Australia; 5 Department of Chemical Pathology, Pathology Queensland, Brisbane, QLD, Australia; 6 Department of Pathology, University of Melbourne, Melbourne, VIC, Australia

## Abstract

Sessile serrated adenomas are the precursor polyp of approximately 20% of colorectal carcinomas. Sessile serrated adenomas with dysplasia are rarely encountered and represent an intermediate step to malignant progression, frequently associated with loss of MLH1 expression. Accurate diagnosis of these lesions is important to facilitate appropriate surveillance, particularly because progression from dysplasia to carcinoma can be rapid. The current World Health Organization classification describes two main patterns of dysplasia occurring in sessile serrated adenomas, namely, serrated and conventional. However, this may not adequately reflect the spectrum of changes seen by pathologists in routine practice. Furthermore, subtle patterns of dysplasia that are nevertheless associated with loss of MLH1 expression are not encompassed in this classification. We performed a morphological analysis of 266 sessile serrated adenomas with dysplasia with concurrent MLH1 immunohistochemistry with the aims of better defining the spectrum of dysplasia occurring in these lesions and correlating dysplasia patterns with MLH1 expression. We found that dysplasia can be divided morphologically into four major patterns, comprising minimal deviation (19%), serrated (12%), adenomatous (8%) and not otherwise specified (79%) groups. Minimal deviation dysplasia is defined by minor architectural and cytological changes that typically requires loss of MLH1 immunohistochemical expression to support the diagnosis. Serrated dysplasia and adenomatous dysplasia have distinctive histological features and are less frequently associated with loss of MLH1 expression (13 and 5%, respectively). Finally, dysplasia not otherwise specified encompasses most cases and shows a diverse range of morphological changes that do not fall into the other subgroups and are frequently associated with loss of MLH1 expression (83%). This morphological classification of sessile serrated adenomas with dysplasia may represent an improvement on the current description as it correlates with the underlying mismatch repair protein status of the polyps and better highlights the range of morphologies seen by pathologists.

Sessile serrated adenomas account for approximately 15% of all endoscopically removed polyps.^1, 2, 3^ They are the precursor of most carcinomas arising via the serrated neoplasia pathway and as such are responsible for 20–30% of all colorectal carcinomas.^4, 5, 6^ Carcinomas of the serrated pathway are overrepresented in studies of interval colorectal carcinomas.^7^ This occurs due to a range of factors, including endoscopically missed precursor lesions, incompletely resected lesions, rapid progression of *de novo* lesions and inadequate surveillance due to pathological misdiagnosis.^8, 9^ Therefore, efforts to improve colonoscopic detection and pathological diagnosis of sessile serrated adenomas should lead to a reduction in interval colorectal carcinomas.

Sessile serrated adenomas progress to carcinoma via an intermediate step of sessile serrated adenoma with dysplasia. These are advanced lesions with a high risk of rapid progression to malignancy.^10, 11^ Histologically, ordinary sessile serrated adenomas are defined by abnormal crypt architecture with dilatation of the crypt bases, excessive luminal serrations and absence of cytological atypia, regardless of lesion size or location.^1^ Sessile serrated adenomas with dysplasia are identified by an abrupt transition from ordinary sessile serrated adenoma to overt dysplasia. At a molecular level, the majority of sessile serrated adenomas harbor a somatic *BRAF* mutation and display the CpG island methylator phenotype.^12^ Transition to dysplasia is associated with methylation-induced silencing of tumor-suppressor genes, one of which is *MLH1*.^13^

The histological criteria for the diagnosis of sessile serrated adenoma with dysplasia are not well described. The 2010 World Health Organization classification distinguishes two dysplasia patterns: dysplasia resembling that of conventional adenomas and serrated dysplasia.^14^ In our practice, we do not believe that this adequately describes the morphological spectrum of sessile serrated adenomas with dysplasia. In particular, we have identified examples of sessile serrated adenomas with very subtle changes that have nevertheless lost expression of MLH1 by immunohistochemistry. Because these lesions have advanced molecular changes, their associated risk of malignant progression is likely to be high, despite the relative lack of cytological abnormalities. Failure to identify this risk by the pathologist may result in inadequate surveillance and thus increases the risk of interval colorectal carcinoma. In addition, we hypothesize that certain patterns of dysplasia predict the underlying molecular features of the lesion, such as mismatch repair protein function. Thus we reviewed a large series of sessile serrated adenomas with dysplasia with the aim of better characterizing the spectrum of dysplasia, in particular sessile serrated adenomas with subtle morphological changes that are nevertheless associated with loss of mismatch repair protein function.

## Materials and methods

### Case Selection

Cases were identified via retrospective database search using the search terms ‘sessile serrated adenoma’ or ‘sessile serrated polyp’ in combination with ‘dysplasia’ or ‘carcinoma’ over a 4-year period (between February 2013 and January 2017) at Envoi Specialist Pathologists, a gastrointestinal pathology practice in Brisbane, Australia. Patient age, gender and lesion anatomical location were obtained from the pathology request form or the colonoscopy report when available. Lesions were classified as proximal (proximal to the splenic flexure) or distal (distal to and inclusive of the splenic flexure). The sample set included lesions removed by polypectomy, endoscopic mucosal resection and colectomy. Lesion size data were obtained from the colonoscopy report. For colectomy specimens, the size was determined as the maximum lesion diameter from histological slides where complete polyps could be visualized. Patients with serrated polyposis syndrome were not excluded. No patients had a diagnosis of familial adenomatous polyposis, Lynch syndrome or inflammatory bowel disease. This study was approved by the ethics committee of QIMR Berghofer Medical Research Institute (P1298).

Hematoxylin and eosin-stained sections of all cases identified using the search terms were retrieved for initial histological review by one pathologist (CL). For inclusion in the study, lesions were required to show a sessile serrated adenoma component with abrupt transition to dysplasia in at least one tissue fragment. The sessile serrated adenoma had to display at least one typical sessile serrated adenoma-type crypt as previously described.^1^ The dysplastic component was defined by a combination of abnormal crypt architecture and cytological atypia. Cases of traditional serrated adenomas arising from sessile serrated adenomas were excluded. As previously reported, traditional serrated adenomas were defined by displaying at least two of the following features: eosinophilic cytoplasm with pencillate nuclei, slit-like epithelial serrations, and ectopic crypt formations.^15^ Cases classified as invasive carcinoma only without residual dysplasia or dysplastic fragments without contiguous sessile serrated adenoma were excluded. Cases with no residual dysplastic component on MLH1 immunohistochemistry were also excluded. Excluded cases were all confirmed after review by three other pathologists (CR, MLB, NIW).

### Histological Review

All included cases of sessile serrated adenomas with dysplasia were reviewed first by one pathologist (CL), who evaluated the range of architectural changes and cytological atypia to classify these lesions into different patterns. A pattern had to involve at least one complete colonic crypt. All cases were subsequently reviewed by the other three pathologists (CR, MLB, NIW), and any disagreements regarding classification were resolved via group consensus.

### MLH1 Immunohistochemistry

Immunohistochemistry for MLH1 was performed on all cases as previously described.^15^ MLH1 staining was assessed individually for each pattern of dysplasia. When more than one dysplasia pattern was present in one lesion, the lesion was considered MLH1 deficient if at least one pattern displayed loss of immunohistochemical MLH1 expression.

### Statistical Analysis

Categorical variables were compared using Fisher’s exact test. Continuous variables were compared using Student’s *t*-test or non-parametric Kruskal–Wallis test for median comparisons. A *P*-value of <0.05 was considered significant.

## Results

### Clinical Data

The final study group comprised 266 sessile serrated adenomas with dysplasia from 230 patients, representing 2% of all sessile serrated adenomas reported during the same period of time. There were 151 (66%) females and 79 (34%) males, with a mean age at diagnosis of 75 years (range 34–97 years). Fourteen lesions were obtained from eight patients with serrated polyposis syndrome. A total of 210 (85%) lesions were from the proximal colon, and 50 (19%) lesions were associated with invasive carcinoma. The median size was 12 mm (range 4–70 mm), with 40% of lesions measuring <10 mm. A subset of these lesions (*N*=123) were used in a previous study by our group.^10^

### Morphological Patterns of Dysplasia in Relation to MLH1 Immunohistochemical Expression

We divided morphological dysplasia into four major patterns (Table 1). The minimal deviation, serrated and adenomatous patterns had specific, distinctive characteristics. All other dysplasia morphologies that did not fall into the aforementioned patterns were classified as dysplasia not otherwise specified, which encompassed a wide range of histological features. A total of 46 (17%) lesions exhibited more than one dysplasia pattern. Overall, MLH1 loss of expression was identified in 193 (73%) of lesions.

#### Minimal deviation dysplasia

**Figure 1:**
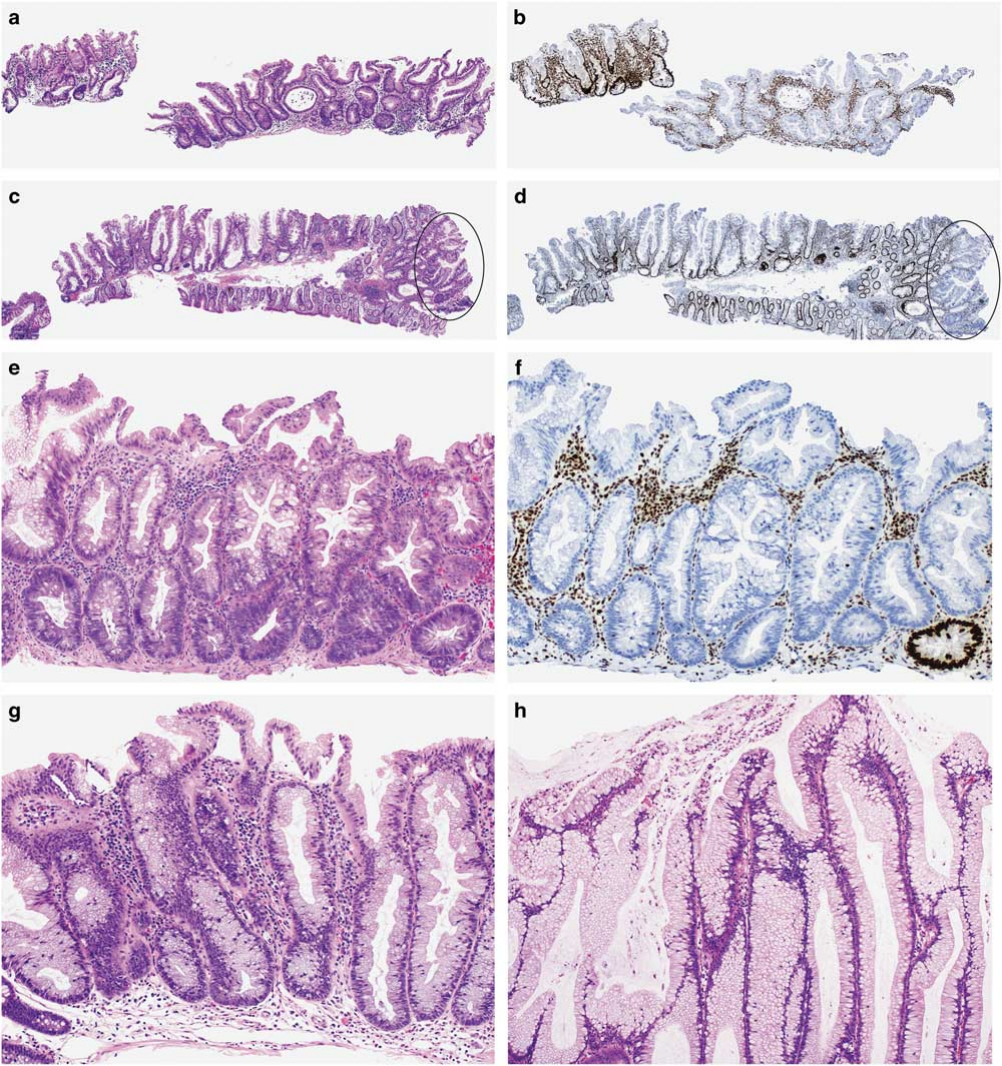

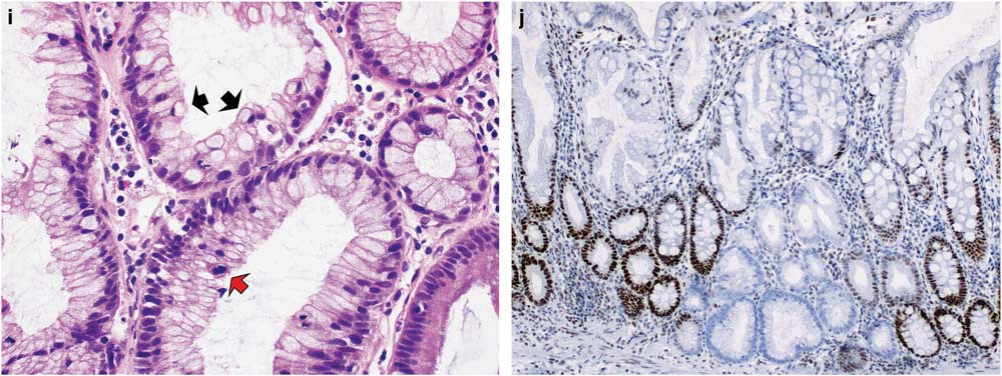
Examples of minimal deviation dysplasia. At low-to-medium magnification, the architectural changes are subtle with mild crowding of crypts separated by less lamina propria and showing some degree of disorganization (circled areas in panels (**c** and **d**)) (**a**, **c** and **e**: hematoxylin and eosin; **b**, **d** and **f**: corresponding MLH1 immunohistochemistry). The cells are hypermucinous with some crowding of nuclei, focal hyperchromasia, loss of polarity, mitotic figures (red arrow, **i**) and dystrophic mucus cells on the surface (black arrow, **i**) (**g**–**i**). Incidental cluster of crypts with MLH1 loss of expression in a lesion showing overt dysplasia elsewhere (**j**).

This pattern was seen in 50 (19%) cases and was the only dysplasia pattern in 14 lesions. It exhibits minimal architectural and cytological changes and is difficult to identify histologically. At low magnification, there is mild crypt disorganization, crypt crowding and reduced luminal serration compared with the background sessile serrated adenoma (Figure 1). The cells frequently have a hypermucinous appearance with compressed, basally located nuclei showing mild hyperchromasia, compared with the nuclei of the adjacent sessile serrated adenoma component. Less commonly, the cytoplasm is mildly eosinophilic with apical mucin. Some nuclei are abnormally located near the lumen of the crypt and display loss of polarity and mitotic figures. Dystrophic mucus cells are sometimes seen on the surface of the dysplastic crypt lining. This pattern was associated with loss of MLH1 expression in 91%. In only seven cases, MLH1 expression was retained with strong MLH1 staining in lesions that had other patterns of dysplasia.

#### Serrated dysplasia

**Figure 2:**
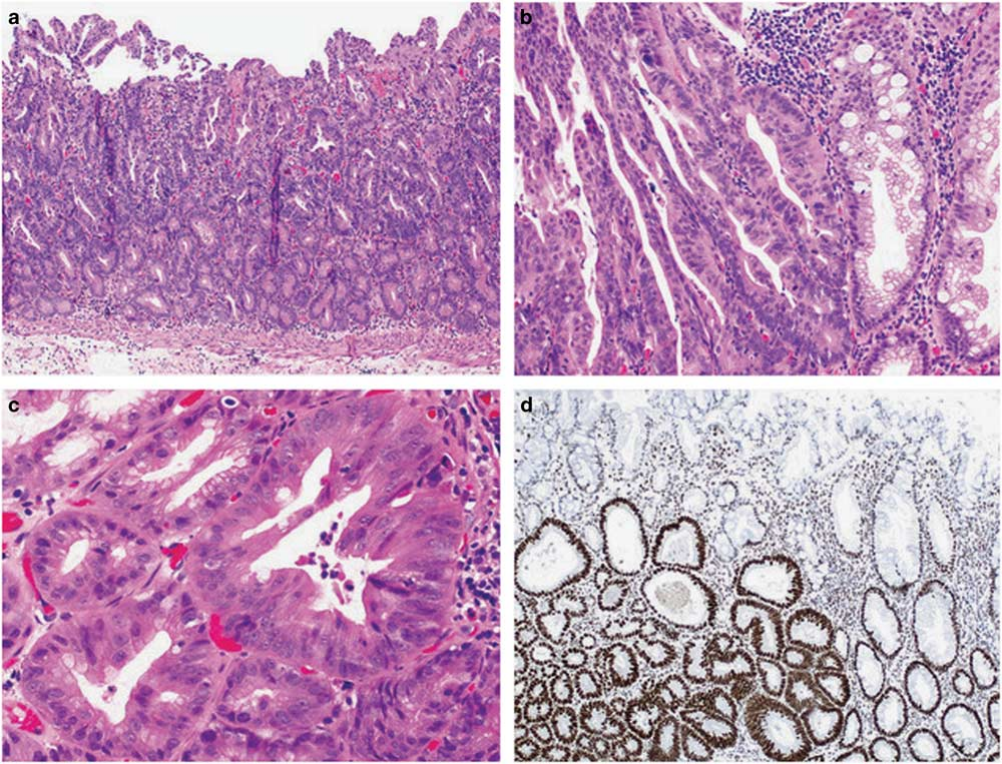
Serrated dysplasia characterized by small packed glandular structures with abundant eosinophilic cytoplasm. The dysplastic nuclei are round and vesicular with often prominent nucleoli (**a**–**c**). The majority of sessile serrated adenomas with serrated dysplasia demonstrate retained MLH1 expression by immunohistochemistry (**d**).

**Figure 3:**
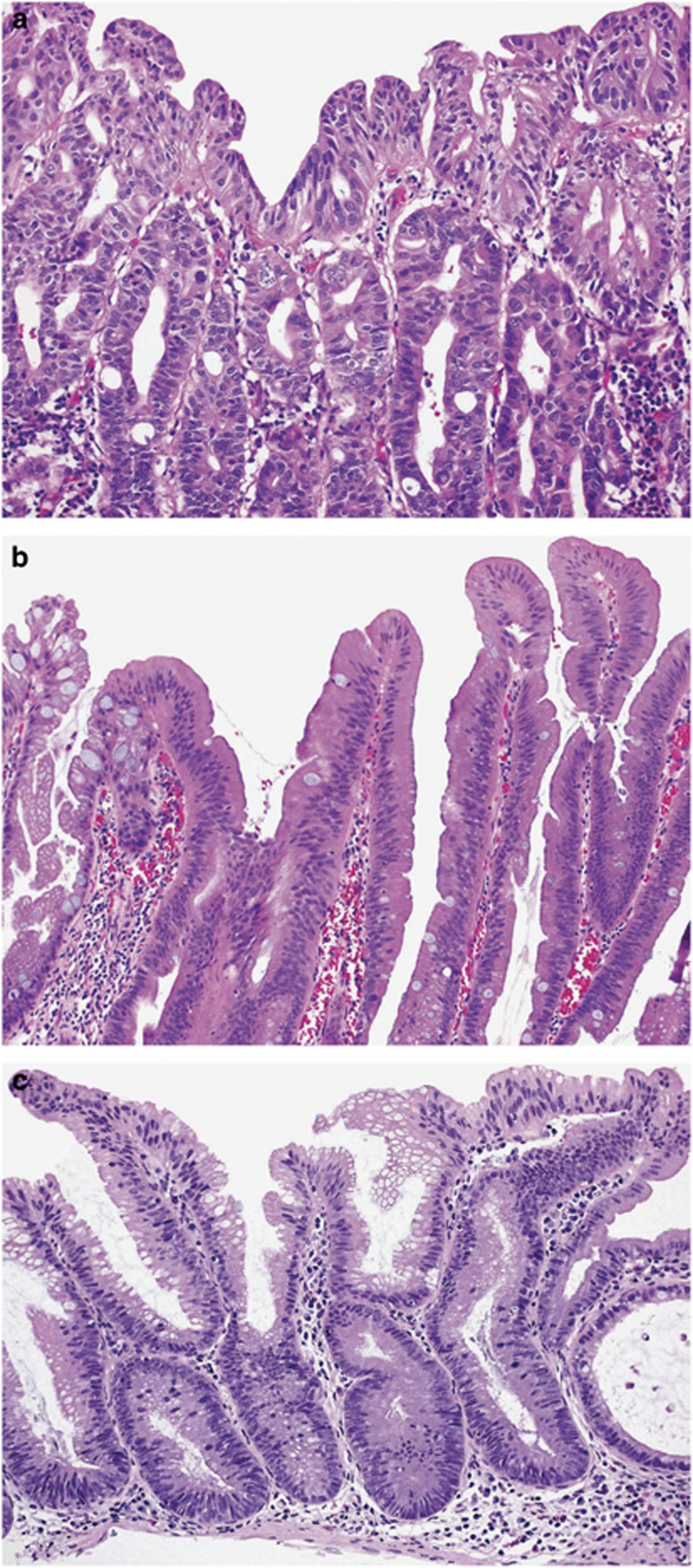
Differences in histological appearances of a sessile serrated adenoma with serrated dysplasia (**a**), a flat traditional serrated adenoma arising from a sessile serrated adenoma (**b**) and a sessile serrated adenoma with minimal deviation dysplasia (**c**). The nuclei of traditional serrated adenomas are pencillate, uniform and basally located compared with round atypical nuclei in serrated dysplasia showing loss of polarity. The cytoplasm in some examples of minimal deviation dysplasia can be partially eosinophilic, but staining is less intense compared with traditional serrated adenoma.

This less common dysplasia pattern was identified in 31 (12%) cases and was the only dysplasia pattern in 22 lesions. An eosinophilic appearance at low power with tightly packed crypts is characteristic (Figure 2). It shows closely packed small glands that occupy the full thickness of the mucosa with occasional cribriform growth. Architectural serration is less prominent. The cells are cuboidal to low columnar with evident dysplasia, containing round vesicular nuclei, prominent nucleoli and abundant eosinophilic cytoplasm. Mitoses are frequent, extend to the luminal surface and can be atypical. Despite the presence of eosinophilic cells, this must be distinguished from flat traditional serrated adenoma arising from a sessile serrated adenoma. Flat traditional serrated adenomas show eosinophilic cells toward the luminal aspect, possess uniform pencillate nuclei and only rare mitotic activity (Figure 3). The serrated dysplasia pattern had a strong predilection to retain staining for MLH1 (loss of MLH1 expression in 13%).

#### Adenomatous dysplasia

**Figure 4:**
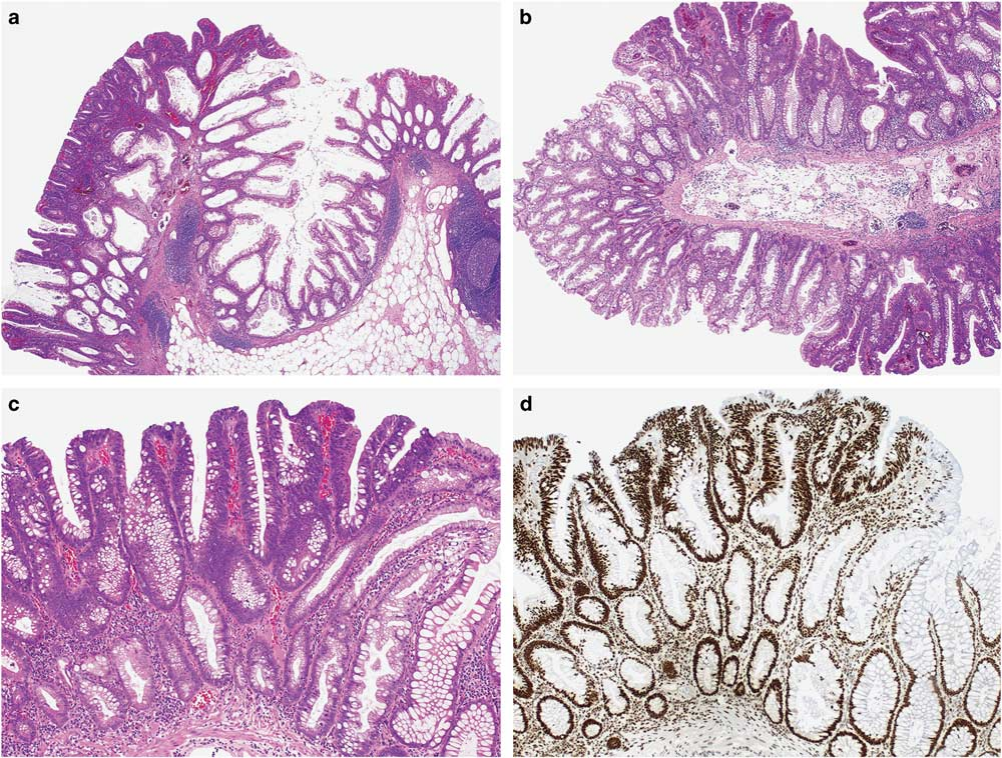
Adenomatous dysplasia is a distinctive pattern with a predominant ‘top–down’ appearance and complete similarity to dysplasia seen in conventional adenomas (**a**–**c**). The majority of lesions retain MLH1 expression by immunohistochemistry (**d**).

This distinctive dysplasia pattern was identified in 21 (8%) cases and was the only dysplasia pattern in all but 1 lesion. The main characteristics of adenomatous dysplasia are the predominant location of the dysplastic component on the surface (‘top–down’ dysplasia) with preserved non-dysplastic sessile serrated adenoma at the base of the lesion and the complete similarity to the dysplasia of conventional adenomas (Figure 4). There is no serration. The cells are columnar with at least focal goblet cell differentiation, elongated nuclei and pseudostratification. This pattern also had a strong predilection to retain staining for MLH1 (loss of MLH1 expression in 5%).

#### Dysplasia not otherwise specified

**Figure 5:**
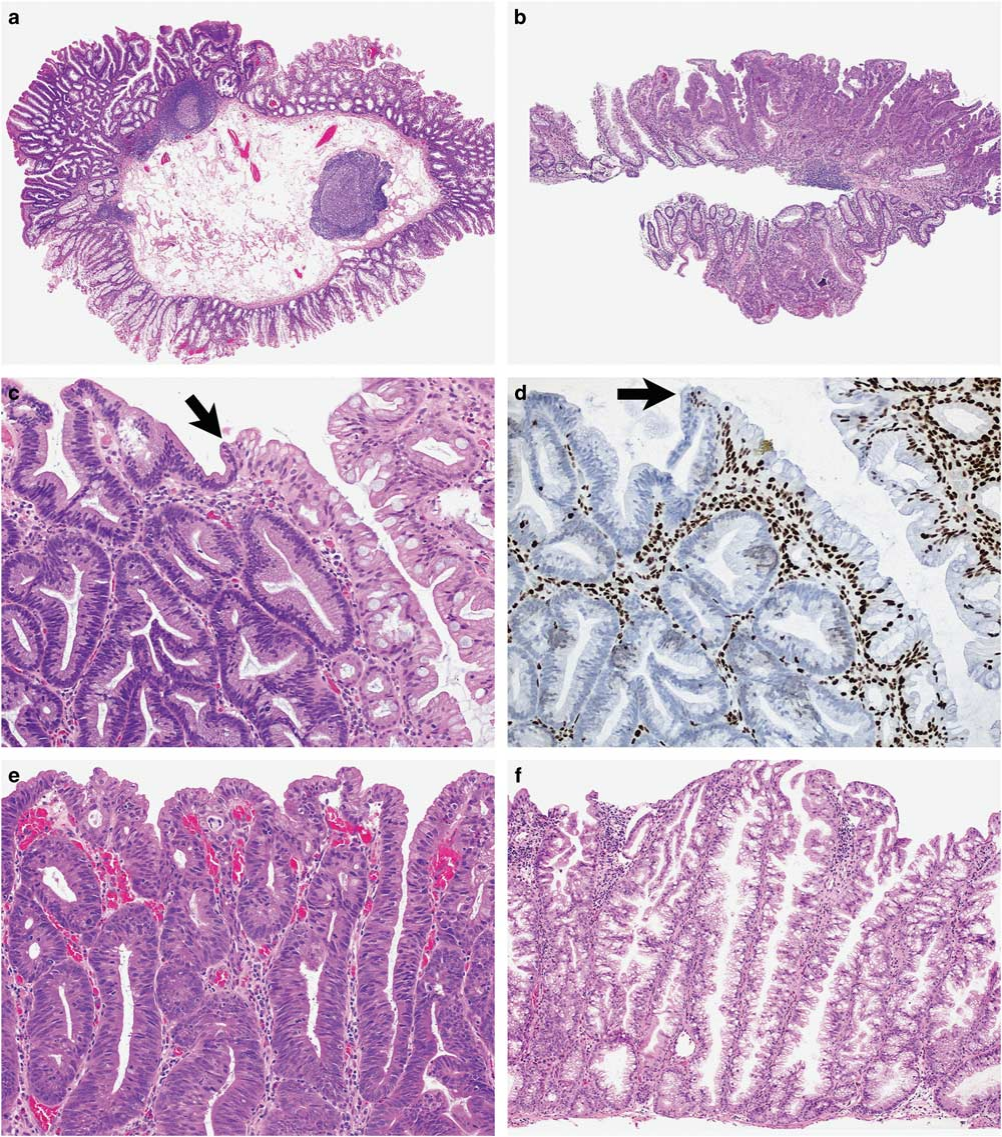

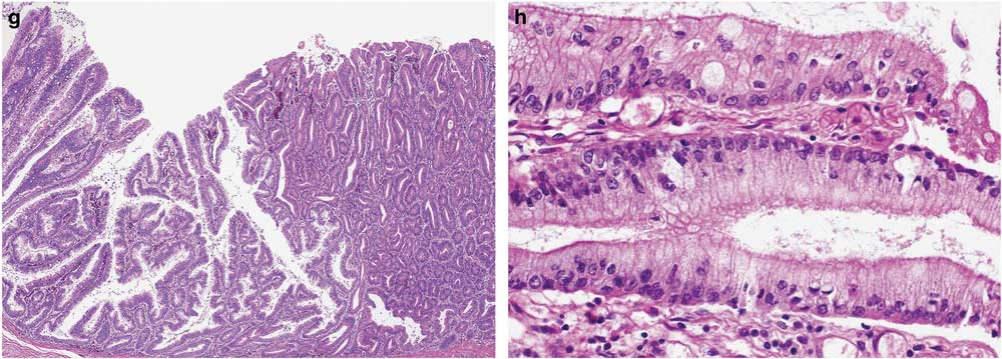
Dysplasia not otherwise specified can present as a protuberant (**a**) or a flat (**b**) lesion at low magnification with an abrupt transition (black arrow) from the non-dysplastic component on hematoxylin and eosin stain (**c**) and MLH1 immunohistochemistry (**d**). The morphological appearance is heterogeneous and can display areas of crypt crowding showing little serration, loss of cytoplasmic mucin and marked cytological atypia (**e**) and areas of elongated crypts with increased serration (**f**), with often more than one architectural pattern in one lesion (**g**). A gastric phenotype is sometimes encountered characterized by foveolar mucin and round nuclei (**h**).

This was the most common pattern, seen in 211 (79%) cases, and included all examples that did not meet the criteria for one of the special patterns of dysplasia described above. As such, the morphological appearances within this pattern are variable, but they encompass the most easily identified and prototypical sessile serrated adenomas with dysplasia. In all cases, there are obvious architectural and cytological abnormalities. Architectural dysplasia includes crypt elongation, crypt crowding, excessive serration and complex branching or cribriform growth (Figure 5). Cytological dysplasia is evident at low magnification and occupies the full thickness of the epithelium. The columnar dysplastic cells have basal hyperchromatic nuclei with pseudostratification, increased mitotic activity and loss of polarity and cytoplasm ranges from eosinophilic to amphophilic. Less commonly, a gastric foveolar appearance is observed, where there is glandular crowding and elongation, and the cells contain pale eosinophilic cytoplasm with apical mucin. Some nuclei are abnormally located near the lumen of the crypt and display loss of polarity with mitotic figures. Despite encompassing a ‘waste-basket’ of patterns of dysplasia, this group was united by a high rate of MLH1 loss of expression (83%).

Significant clinicopathological differences were identified when specific dysplasia patterns were compared with dysplasia not otherwise specified (Table 1). Minimal deviation dysplasia was most frequently associated with other dysplastic patterns, essentially with dysplasia not otherwise specified. Compared with dysplasia not otherwise specified, serrated dysplasia was associated with younger age at diagnosis (71 *versus* 77 years, *P*=0.001), distal location (29 *versus* 12%, *P*=0.03) and retained MLH1 staining (87 *versus* 17%, *P*<0.001). Adenomatous dysplasia was more frequently diagnosed in younger patients (72 *versus* 77 years, *P*=0.03), with frequent retained MLH1 staining (95 *versus* 17%, *P*<0.001) and less frequently associated with invasive carcinoma (0 *versus* 22%, *P*=0.01). No significant difference in median lesion size was found between dysplastic patterns.

In addition to the patterns of dysplasia described above, we incidentally identified single or small clusters of crypt bases with loss of MLH1 expression within ordinary sessile serrated adenomas (Figure 1j). They were seen in 86 (32%) of 266 lesions and always associated with another dysplasia pattern elsewhere. We did not regard this as a distinct pattern of dysplasia as it could not be identified on routine sections.

## Discussion

Sessile serrated adenomas with dysplasia have been reported in the gastroenterology literature as representing ‘triple threat’ precursors for interval colorectal carcinomas.^16^ These lesions are rapidly progressive, difficult to detect endoscopically and commonly incompletely resected. However, we feel that a ‘quadruple threat’ may be more appropriate, with the addition of pathological misdiagnosis. To gain insight into the range of morphological patterns of dysplasia in sessile serrated adenomas, we studied 266 lesions and reported the characteristics of four patterns of dysplasia: (1) minimal deviation dysplasia in 19% of lesions characterized by subtle architectural and cytological changes and often identified with the help of MLH1 loss of expression; (2) serrated dysplasia in 12% of lesions associated with retained MLH1 expression; (3) adenomatous dysplasia in 8% of lesions characterized by a ‘top–down’ appearance resembling dysplasia in conventional adenoma and retained MLH1 expression; and (4) dysplasia not otherwise specified, seen in 79% of lesions and the most common pattern, encompassing a wide range of morphological changes and usually MLH1 deficient.

At the inception of this study, minimal deviation dysplasia was not recognized as a distinct entity. The existence of this type of dysplasia was suggested by the observation that MLH1 loss in sessile serrated adenomas with dysplasia sometimes encompassed not only the evidently dysplastic focus but also adjacent areas not readily recognizable as dysplastic on routine sections. As we became familiar with the subtle changes associated with this pattern, we were able to identify it in a significant proportion of sessile serrated adenomas with dysplasia, usually associated with another dysplasia pattern and MLH1 loss. Minimal deviation dysplasia was commonly associated with other more obvious dysplasia patterns; however, it was the only dysplasia pattern in 14 lesions. Minimal deviation dysplasia with retained MLH1 expression is difficult to diagnose. In our series, seven cases all associated with other dysplasia patterns showed minimal deviation dysplasia with retained MLH1 expression. More studies are required to assess the reproducibility of this diagnosis and to provide evidence at the molecular level that minimal deviation with retained MLH1 expression represents progression from a sessile serrated adenoma. Until then, we recommend that minimal deviation dysplasia should be defined by a combination of subtle morphological changes and concurrent loss of MLH1 expression. When there is no other pattern present, we require MLH1 loss for the diagnosis. Identification of this pattern is important as it can be the only dysplasia pattern present in a specimen from more heterogeneous lesions that have not been completely resected.

Possibly representing the extreme manifestation of minimal deviation dysplasia, MLH1 loss can also occur incidentally in occasional crypt bases from otherwise morphologically typical sessile serrated adenomas. This was identified in 32% of our cases and always associated with more extensively dysplastic components in other areas of the lesion. Because there are no morphological clues to identify these cases, they are at present a curiosity that can only be recognized when MLH1 staining is performed for other reasons. Such incidental MLH1 loss in sessile serrated adenomas had been noted and illustrated previously,^17, 18, 19, 20^ where it was regarded as due to *MLH1* promoter methylation prior to development of histological dysplasia. In Lynch syndrome, mismatch repair-deficient crypt foci had also been reported in the normal mucosa of resection specimens for colorectal carcinomas.^21^ The significance of these small mismatch repair-deficient foci is unclear. In sessile serrated adenomas that already harbor the somatic *BRAF* mutation and CpG island methylator phenotype, the occurrence of mismatch repair deficiency by somatic methylation of *MLH1* may signify an advanced lesion, despite the absence of morphological abnormalities. More studies are needed to determine the frequency and the significance of such foci ‘caught in the act of methylation’, when isolated MLH1-deficient crypts occur in sessile serrated adenomas without obvious dysplasia. In practice, we do not regard these foci as dysplastic as they cannot be identified on routine sections.

Both serrated dysplasia and the adenomatous dysplasia have distinctive morphological features easy to recognize in routine stains, identified in <20% of lesions and usually as the only dysplasia pattern. Nonetheless, despite their rarity, serrated dysplasia and adenomatous dysplasia should be recognized as they are largely MLH1 retained. Therefore, sessile serrated adenomas with serrated or adenomatous dysplasia may be the precursor lesions of *BRAF*-mutant mismatch repair-proficient cancers, the most aggressive molecular subgroup of colorectal carcinomas.^22, 23^ Compared with dysplasia not otherwise specified, the adenomatous dysplasia is more pronounced at the top of the lesion and is uniform in appearance, resembling dysplasia in conventional adenomas. In equivocal cases, MLH1 immunohistochemistry may be useful.

The histological heterogeneity of sessile serrated adenomas with dysplasia had been illustrated in earlier studies.^24, 25, 26, 27^ Our major group of dysplasia not otherwise specified exhibits a broad spectrum of architectural changes, including crowding, hyperserration and cribriform growth, while cytological changes include mucin-depleted, foveolar and hypermucinous forms. It is unclear why sessile serrated adenomas with dysplasia display such marked heterogeneity, especially when compared with conventional adenomas, which are histologically more uniform. One possible explanation is a consequence of the CpG island methylator phenotype, where the progressive methylation of multiple gene promoters offers a continuous gradient of protein expression resulting in diverse histological changes. In cases with MLH1 loss of expression, the superimposed hypermutator phenotype may lead to further genetic aberrations and morphological variability.

Differential diagnosis of sessile serrated adenoma with dysplasia includes non-dysplastic atypia and traditional serrated adenomas. Regenerative changes secondary to inflammation, prolapse-related crypt distortion and cross-cut sections through basal crypts can mimic dysplasia in sessile serrated adenomas. In these cases, there is a gradation of changes rather than an abrupt transition, and the crypts maintain evenly spaced. Prolapse-related distortion results in crypt compression with an angulated, hyperchromatic appearance toward the base, associated with lamina propria fibrosis and smooth muscle proliferation. However, orderly maturation toward the luminal aspect is preserved. Serrated dysplasia must be distinguished from flat traditional serrated adenoma arising within sessile serrated adenoma (Figure 3). Although both entities have abundant eosinophilic cytoplasm, traditional serrated adenoma is mostly superficial, has small pencillate nuclei without atypia and infrequent or absent mitoses.

In routine practice, the most important point for pathologists is to recognize the different patterns of dysplasia that can be seen in sessile serrated adenomas, rather than using this proposed classification in the pathology report. We do not recommend grading dysplasia, as the myriad architectural and cytological features renders this poorly reproducible, and several patterns can be present in a single case. Furthermore, *MLH1* methylation with loss of immunohistochemical expression is the most critical molecular event underpinning lesion progression, present not only in histologically obvious dysplasia but also in sessile serrated adenomas with dysplasia displaying very subtle morphological changes. Categorizing these lesions as low grade would convey the wrong message to clinicians that they are innocuous. When reporting sessile serrated adenomas with dysplasia, regardless of the morphological patterns present, we recommend emphasizing the advanced nature of the lesion and the need for short-term follow-up endoscopy to verify that the lesion has been completely excised.

MLH1 immunohistochemistry is a useful ancillary test to support the diagnosis of dysplasia in sessile serrated adenomas in some situations but should not be performed on every lesion. In sessile serrated adenomas with unequivocal architectural and cytological dysplasia, MLH1 expression status does not alter the final diagnosis. It is recommended in the following situations: (1) equivocal cytological atypia possibly secondary to inflammation, prolapse or cross-cut of the crypt bases; (2) piecemeal resection with separate fragments of dysplasia and sessile serrated adenoma, where loss of MLH1 expression in the dysplastic fragments favors sessile serrated adenoma with dysplasia rather than conventional adenoma admixed with sessile serrated adenoma; and (3) lesions with mild morphological changes of minimal deviation dysplasia to support the diagnosis of dysplasia. We initially used MLH1 loss as a training tool to recognize minimal deviation dysplasia and still perform MLH1 immunohistochemistry when only subtle morphological changes are present.

In summary, sessile serrated adenomas with dysplasia are heterogeneous lesions often exhibiting multiple different dysplasia patterns. The most significant finding of this study is the recognition of minimal deviation dysplasia. The accurate diagnosis of these subtle lesions is challenging for pathologists and MLH1 expression should be used when suspected on routine stains. Serrated dysplasia and adenomatous dysplasia are rarely encountered and account for most of lesions with retained MLH1 expression. Currently, all these dysplastic patterns are reported as sessile serrated adenoma with dysplasia, the only entity recognized by the latest World Health Organization classification. The heterogeneity of these advanced lesions and the distinctive features of some dysplastic patterns, in particular the serrated pattern, may justify expanding the current classification of serrated polyps to account for these different subgroups. Further studies are needed to assess the reproducibility of diagnosing the different dysplastic patterns. Better awareness of the histological spectrum of sessile serrated adenomas with dysplasia will increase diagnostic accuracy and may reduce the incidence of interval colorectal carcinomas.

## Figures and Tables

**Table 1:** Clinicopathological characteristics of patterns of dysplasia

*Dysplasia pattern*	*Total*	*Patient age, years*	*Proximal colonic location*	*Polyp size, mm*	*MLH1 loss of expression*	*Association with other patterns*	*Association with invasive carcinoma*
	N *(%)*	*Mean (range)*	N *(%)*	*Median (range)*	N *(%)*	N *(%)*	N *(%)*
Minimal deviation	50 (19)	76 (44–86)	43 (90)	11.5 (5–55)	43 (91)	36 (72)^a^	4 (8)
Serrated	31 (12)	71 (35–89)^a^	20 (71)^a^	9 (5–20)	4 (13)^a^	9 (29)	4 (13)
Adenomatous	21 (8)	72 (36–89)^a^	15 (75)	15 (6–27)	1 (5)^a^	4 (19)	0 (0)^a^
Not otherwise specified	211 (79)	77 (34–97)	174 (88)	13 (4–70)	175 (83)	44 (21)	47 (22)
All lesions	266 (100)	75 (34–97)	210 (85)	12 (4–70)	193 (73)	46 (17)	50 (19)

a Indicates significance difference (*P*<0.05) when compared with characteristic of dysplasia not otherwise specified.
